# Comparative Histological and Histomorphometric Results of Six Biomaterials Used in Two-Stage Maxillary Sinus Augmentation Model after 6-Month Healing

**DOI:** 10.1155/2018/9430989

**Published:** 2018-06-27

**Authors:** Gerardo La Monaca, Giovanna Iezzi, Maria Paola Cristalli, Nicola Pranno, Gian Luca Sfasciotti, Iole Vozza

**Affiliations:** ^1^Department of Sense Organs, Sapienza University of Rome, Rome, Italy; ^2^Department of Medical, Oral and Biotechnological Sciences, University of Chieti-Pescara, Chieti Scalo, Italy; ^3^Department of Biotechnologies and Medical Surgical Sciences, Sapienza University of Rome, Rome, Italy; ^4^Department of Oral and Maxillofacial Sciences, Sapienza University of Rome, Rome, Italy

## Abstract

**Objectives:**

To evaluate the performances of six different bone substitute materials used as graft in maxillary sinus augmentation by means of histological and histomorphometric analysis of bone biopsies retrieved from human subjects after a 6-month healing period.

**Materials and Methods:**

Six consecutive patients (3 males, 3 females, aged 50-72 years), healthy, nonsmokers, and with good oral hygiene, presenting edentulous posterior maxilla with a residual bone crest measuring ≤ 4 mm in vertical height and 3 to 5 mm in horizontal thickness at radiographic examination, were selected to receive sinus augmentation and delayed implant placement. Under randomized conditions, sinus augmentation procedures were carried out using mineralized solvent-dehydrated bone allograft (MCBA), freeze-dried mineralized bone allograft (FDBA), anorganic bovine bone (ABB), equine-derived bone (EB), synthetic micro-macroporous biphasic calcium-phosphate block consisting of 70% beta-tricalcium phosphate and 30% hydroxyapatite (HA-*β*-TCP 30/70), or bioapatite-collagen (BC). After 6 months, bone core biopsies were retrieved and 13 implants were placed. Bone samples were processed for histological and histomorphometric analysis. CT scans were taken before and after surgery. After 4 months of healing, patients were restored with a provisional fixed acrylic resin prosthesis, as well as after further 2-4 months with a definitive cemented zirconia or porcelain-fused-to-metal crowns.

**Results:**

There were no postoperative complications or implant failures. The histological examination showed that all biomaterials were in close contact with newly formed bone, surrounding the graft granules with a bridge-like network. No signs of acute inflammation were observed. The histomorphometry revealed 20.1% newly formed bone for MCBA, 32.1% for FDBA, 16.1% for ABB, 22.8% for EB, 20.3% for HA-*β*-TCP 30/70, and 21.4% for BC.

**Conclusions:**

Within the limitations of the present investigation, all the six tested biomaterials showed good biocompatibility and osteoconductive properties when used in sinus augmentation procedures, although the FDBA seemed to have a better histomorphometric result in terms of newly formed bone and residual graft material. This trial is registered with ClinicalTrials.gov Identifier (Registration Number): NCT03496688.

## 1. Introduction

The lack of adequate bone height and thickness negatively affects implant-supported rehabilitation in the edentulous posterior maxilla. Therefore, bone-grafting procedures are needed to increase the available bone volume and to provide structural and mechanical support for the placement of dental implants.

Among graft materials, autologous bone is considered the gold standard due to its osteogenic, osteoinductive, and osteoconductive properties [1–3]. However, the use of autogenous bone has significant drawbacks such as a limited intraoral supply, the need of general anesthesia in case of extraoral harvesting, donor site morbidity, increased operating time, need of two surgical sites, tendency to partial resorption and potential intraoperative, and postoperative complications [2, 4–7].

To overcome these disadvantages, a large number of biomaterials have been used alone or in combination with autografts in augmentation procedures [8–19]. Among the osteoconductive materials, allografts (fresh-frozen bone, freeze-dried bone, demineralized freeze-dried bone), xenografts (of bovine, equine, or porcine origin), and alloplastic materials (different combination of calcium-phosphate, bioactive glasses, polymers) were described in the dental literature as being able to enhance bone formation. Furthermore, several studies have shown that the biomaterials may not adversely influence clinical outcomes and implant survival when compared to autogenous bone [20, 21].

The two-stage sinus lift augmentation with delayed implant insertion was considered a good clinical model to evaluate the performance of graft materials, because bone formation occurs within an enclosed space and with a minimal interference from external factors. In addition, this procedure is highly predictable and allows collecting bone biopsy specimens during implant insertion avoiding any additional discomfort for the patients [17, 20].

The aim of the present study was to evaluate the performances of six different bone substitute materials used as graft in maxillary sinus augmentation, by means of histological and histomorphometric analysis of bone biopsies retrieved from human subjects after a healing period of 6 months.

## 2. Materials and Methods

### 2.1. Patient Selection

Six patients (3 males, 3 females, aged 50–72 years) who were healthy, nonsmokers, and with good oral hygiene were recruited in this study among those referred to Department of Oral and Maxillofacial Sciences, Sapienza University of Rome, for implant-supported rehabilitation in the posterior atrophic maxilla (Table 1). Inclusion criteria were maxillary partial edentulism in the premolar/molar areas, with a residual bone crest measuring ≤ 4 mm in vertical height and 3 to 5 mm in horizontal thickness as measured on computerized tomography (CT) scan. Exclusion criteria were being pregnant or lactating females, patients with impaired systemic conditions, smoking habit, and maxillary sinus pathology. After clinical and radiographic evaluation, the patients signed a written informed consent form to study participation.

All the clinical procedures were performed in accordance with the Declaration of Helsinki and the Good Clinical Practice Guidelines. The protocol of the study was approved by the Ethical Committee of the Sapienza University of Rome (n. 3447).

### 2.2. Surgical and Restorative Procedures

The preoperative antibiotic and analgesic therapy with Amoxicillin 875 mg + Clavulanic acid 125 mg (Augmentin, GlaxoSmithKline, Belgium) and Ketoprofene 200 mg (Ibifen, 200 mg, IBI Lorenzini, Aprilia, Italy) was given orally 1 hour prior to surgery. Immediately prior to surgery, patients rinsed with a chlorhexidine digluconate solution 0.2% (Corsodyl, GlaxoSmithKline, Belgium) for 2 min, to be continued for 2 weeks postoperatively.

**Figure 1:**
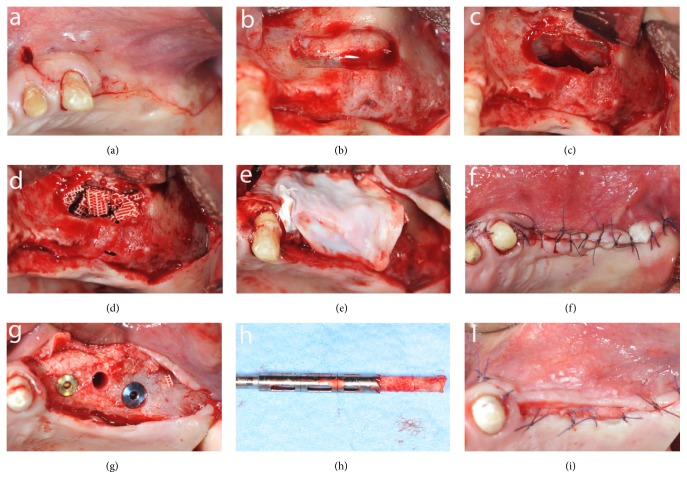
Intraoperative views of the sinus augmentation procedure: (a) mobilization of the mucoperiosteal flap; (b) oval-shaped bony window; (c) sinus membrane elevation; (d) graft material in place; (e) resorbable membrane over the lateral window; (f) suture; (g) bone core biopsy and implant placement; (h) trephine bur and harvested specimen; (i) suture.

Surgery was performed under sterile conditions and local anesthesia (mepivacaine 2% with epinephrine 1:100.000, Carbocaine, AstraZeneca, Italy). A lateral window technique was used for sinus floor elevation. A slightly palatal crestal incision and two vertical releasing incisions were made mesial and distal on the buccal mucosa according to the sinus anatomy to elevate a mucoperiosteal flap (Figure 1(a)). On the lateral side of the sinus wall, the oval-shaped bony window was performed, with the inferior border about 5 mm from the alveolar crest and the superior portion left intact, to create a trapdoor effect (Figure 1(b)). The sinus membrane was carefully raised and, together with the bony window, was rotated inward and upward (Figure 1(c)). The subantral cavity was packed with the graft material (Figure 1(d)) and a resorbable membrane (Bio-Gide, Geistlich Biomaterials Italy S.r.l.) was placed over the lateral wall defect (Figure 1(e)). The mucoperiosteal flap was replaced and stabilized with resorbable interrupted sutures (5-0 Vicryl, Johnson & Johnson Medical, Norderstedt, Germany), which were removed after 2 weeks (Figure 1(f)). Postoperatively, the antibiotic therapy was prescribed for 1 week (Amoxicillin 875 mg + Clavulanic acid 125 mg twice a day) and, if necessary, the analgesic therapy was continued with Ketoprofene 200 mg.

After 6 months clinical and radiographic examinations were performed and each patient was reappointed for biopsy and implant placement in the same location. Under local anesthesia, a full thickness flap was raised and a bone biopsy was performed using a 3.5 mm trephine bur under sterile saline solution irrigation, guided by the radiographic/surgical template in the selected implant site. A total of six bone samples were retrieved from the occlusal aspect of the alveolar crest, one from each augmented site and at least two implants (NobelParallel CC or NobelSpeedy, Nobel Biocare Italiana S.r.l., Italy), were placed according to the manufacturer's indications (Figures 1(g), 1(h), and 1(i)).

To identify crestal bone during histologic and histomorphometric procedures, the harvested specimens were marked with toluidine blue stain on the occlusal side.

After 4 months of healing, patients were provisionally restored with a fixed acrylic resin prosthesis and after 2 to 4 months of function, the definitive prosthetic rehabilitation was applied with cemented zirconia or porcelain-fused-to-metal crowns.

Under randomized conditions, each sinus augmentation procedure was carried out using one of the following six commercial bone substitute materials: mineralized solvent-dehydrated bone allograft (MCBA, Puros®; Zimmer Dental GmbH, Freiburg, Germany); freeze-dried mineralized bone allograft (FDBA, Organizzazione Toscana Trapianti, Azienda Ospedaliero-Universitaria Careggi, Florence, Italy); anorganic bovine bone (ABB, Bio-Oss®, Geistlich Biomaterials Italia S.r.l.); equine-derived bone ( EB osteOXenon®- Bioteck S.p.A., Arcugnano (VI), Italy); synthetic micro-macroporous biphasic calcium-phosphate block consisting of 70% beta-tricalcium phosphate and 30% hydroxyapatite (HA-*β*-TCP 30/70, BioCer Entwicklungs GmbH, Bayreuth, Germany); and bioapatite-collagen ( BC, Biostite™, GABA Vebas San Giuliano Milanese, MI, Italy).

MCBA is cancellous or cortical mineralized solvent-dehydrated bone allograft obtained from cadaveric bone by a processing technique (Tutoplast Process, RTI Biologics, Alachua, FL), which preserves the bone architecture maintaining its biomechanical properties and minimizing antigenicity and infective potential [8, 9, 22].

FDBA is freeze-dried mineralized bone allograft processed using lyophilization; it maintains both the organic and the inorganic component (salts of calcium and phosphate), and when used as a graft material, the mineral content is broken down by osteoclasts, becoming osteoinductive proteins available to induce new bone formation. However, to release osteoinductive proteins from the FDBA organic matrix, a prolonged osteoclast mediated demineralization is needed [23].

ABB is a xenogenic material formed by deproteinized sterilized bovine cancellous bone with 75% porosity and a crystal size of about 10 *μ*m in the form of granules. Its native crystal-line structure is chemically and physically highly similar to human bone and its porous nature promotes the initial biologic processes of cell adhesion and proliferation [19]. This material is well documented and has been shown to be well integrated into host bone tissue in different clinical and histological results [24–26].

EB is an equine-derived bone tissue deantigenated by a proteolytic low temperature process that preserves type 1 bone collagen and makes it anorganic although it conserves unaltered its mineral structure of hydroxyapatite saving the resorption potential [10, 27–29].

HA-*β*-TCP 30/70 is a new bioceramic with reticular structure, which seems to have a better resorption and an increased bone formation due to the levels of released calcium and phosphorous ions able to stimulate new bone formation [8, 16, 30–33]. Indeed, HA seems to act as scaffold and TCP as the resorbable component.

BC is hydroxyapatite associated with type I bovine collagen plus glucosamine. Different studies showed its efficacy as human bone substitute material in the sinus augmentation procedure [34, 35]. The presence of collagen accelerates fibrin formation of the clot while glucosamine improves the bone mineralization progression.

### 2.3. Histological Procedure

The bone cores were retrieved and were immediately stored in 10% buffered formalin and processed to obtain thin ground sections. The specimens were processed using the Precise 1 Automated System (Assing, Rome, Italy) [35]. The specimens were dehydrated in a graded series of ethanol rinses and embedded in a glycol methacrylate resin (Technovit 7200 VLC, Kulzer, Wehrheim, Germany). After polymerization, the specimens were sectioned, along their longitudinal axis, with a high precision diamond disk at about 150 *μ* m, and ground down to about 30 *μ*m with a specially designed grinding machine Precise 1 Automated System (Assing, Rome, Italy). Three slides were obtained from each specimen. These slides were stained with acid fuchsin and toluidine blue and examined with transmitted light Leitz Laborlux microscope (Leitz, Wetzlar, Germany).

Histomorphometry of the percentages of newly formed bone, residual grafted material, and marrow spaces was carried out using a light microscope (Laborlux S, Leitz, Wetzlar, Germany) connected to a high-resolution video camera (3CCD, JVCKY-F55B, JVC, Yokohama, Japan) and interfaced with a monitor and PC (Intel Pentium III 1200 MMX, Intel, Santa Clara, CA, USA). This optical system was associated with a digitizing pad (Matrix Vision GmbH, Oppenweiler, Germany) and a histometry software package with image capturing capabilities (Image-Pro Plus 4.5, Media Cybernetics Inc., Immagini & Computer Snc, Milan, Italy).

## 3. Results

### 3.1. Clinical Results

**Figure 2:**
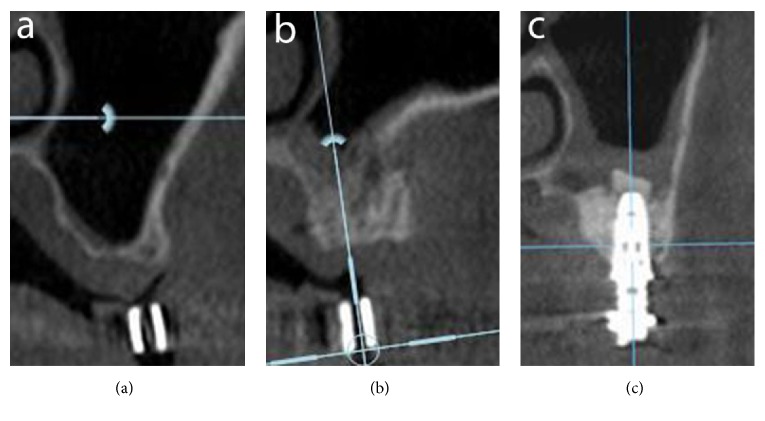
Radiographic evaluation: (a) CT scan before surgery; (b) CT scan after 6 months of graft healing; (c) CT scan after implant placement.

The healing process after sinus augmentation procedures was uneventful. No postoperative complications were present. In no case there was perforation of the sinus membrane. No clinical sign of sinus pathology was observed. Six months after sinus augmentation, the radiographic evaluation of all patients showed the presence of dense bone in the maxillary sinuses where the biomaterials were inserted (Figure 2).

Primary stability of the implants was achieved in all cases independently of the use of bone substitute material (insertion torque value was at least 35 N ). All 13 implants placed during the biopsy retrievement had no complications and were osseointegrated at the end of prosthetic rehabilitation. No failures and no dropouts occurred.

### 3.2. Histological and Histomorphometric Results

**Figure 3:**
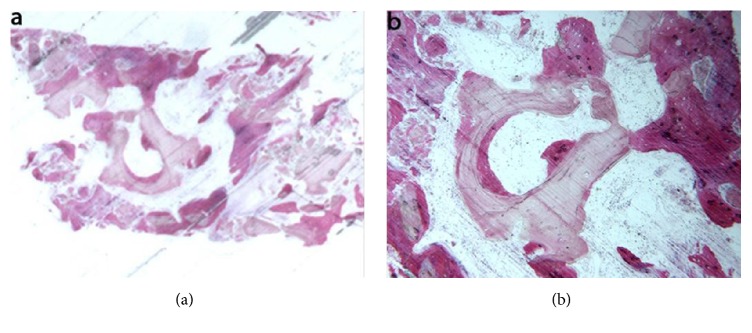
Mineralized solvent-dehydrated bone (toluidine blue and acid fuchsin): (a) trabecular bone with large marrow spaces and biomaterial particles was observed (original magnification 12X); (b**) **the biomaterial particles showed different sizes and they were partially surrounded by newly formed bone that was characterized by large osteocyte lacunae (original magnification 40X).

Mineralized solvent-dehydrated bone (MCBA). At low magnification, trabecular bone with large marrow spaces and biomaterial particles was observed (Figure 3(a)). The biomaterial particles showed different sizes and they were partially surrounded by newly formed bone. Newly formed bone was characterized by large osteocyte lacunae and bridged up greatest part of the biomaterial particles (Figure 3(b)). In some fields, osteoblasts were observed in the process of apposing bone directly on the particle surface. In the marrow spaces only few inflammatory cells were detected. Histomorphometry showed that newly formed bone represented 20.1%, marrow spaces 57.5% and the residual graft material 22.4%.

**Figure 4:**
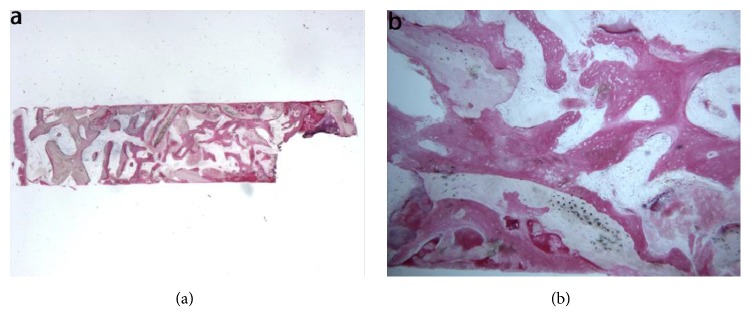
Freeze-dried mineralized bone allograft (toluidine blue and acid fuchsin): (a) newly formed bone with marrow spaces and particles of residual biomaterial was present. In a marginal portion of the sample, preexisting bone with small remodeling areas could be observed (original magnification 12X); (b) the biomaterial particles, showing areas of bone neoformation in their inner part, could be observed. Some of the biomaterial particles showed irregular margins, typical of a resorption process (original magnification 40X).

Freeze-dried mineralized bone allograft (FDBA). At low power magnification, newly formed bone with marrow spaces and particles of residual biomaterial was present. In a marginal portion of the sample, preexisting bone with small remodeling areas could be observed (Figure 4(a)). At high power magnification, in some fields, the biomaterial particles were completely osseointegrated and areas of bone neoformation could be observed also inside the particles. Some of the biomaterial particles showed irregular margins, typical of a resorption process (Figure 4(b)). Bone neoformation areas could be seen both in contact with the biomaterial particles and in the marrow spaces, where few spindle cells could also be detected. Histomorphometry showed that newly formed bone represented 32.1%, marrow spaces 47.8%, and the residual graft material 20.1%.

**Figure 5:**
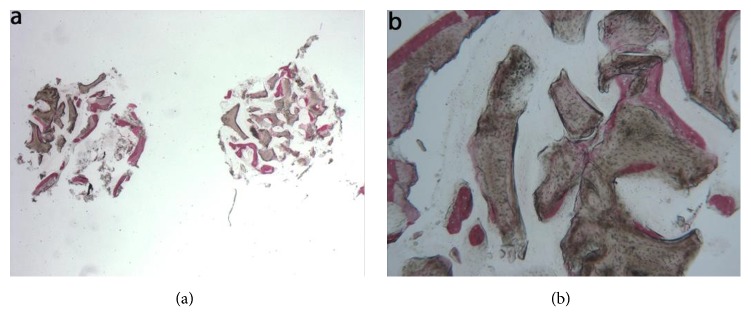
Anorganic bovine bone (toluidine blue and acid fuchsin): (a) the specimen appeared to be constituted by two separate fragments, where several particles of residual biomaterial were evident (original magnification 12X); (b) the areas of bone neoformation in tight contact with the biomaterial surface were present. In some fields, new bone formation inside the biomaterial particles could be observed (original magnification 40X).

Anorganic bovine bone (ABB). At low power magnification, the specimen appeared to be formed by two separate fragments, each presenting several residual biomaterial particles (Figure 5(a)). At high power magnification, most of the biomaterial particles showed areas of bone neoformation in tight contact with the biomaterial surface (Figure 5(b)). The newly formed bone in contact with the biomaterial particles showed wide osteocyte lacunae, typical of a young bone. In some fields, new bone formation inside the biomaterial particles could be observed. Histomorphometry showed that newly formed bone represented 16.1%, marrow spaces 46.7% and the residual biomaterial 37.2%.

**Figure 6:**
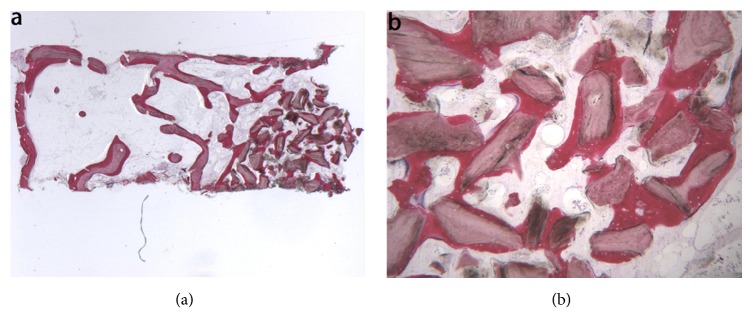
Equine-derived bone (toluidine blue and acid fuchsin): (a) trabecular bone with large marrow spaces and biomaterial particles was observed. The biomaterial particles were located in the apical portion of the biopsy and they were surrounded by new bone (original magnification 12X); (b) the bone was in close contact with the granules and in some areas osteoblasts were observed in the process of apposing bone directly on the particle surface (original magnification 40X).

Equine-derived bone (EB). At low magnification, trabecular bone with large marrow spaces and biomaterial particles was observed (Figure 6(a)). The particles were located in the apical portion of the biopsy and they were surrounded by new bone. In many fields the bone was in strict contact with the granules and in some areas osteoblasts were observed in the process of apposing bone directly on the particle surface (Figure 6(b)). Many large vessels could be detected. No inflammatory cells, or multinucleated giant cells, were present around the biomaterial or at the interface with bone. Histomorphometry showed that newly formed bone represented 22.8%, marrow spaces 47.1%, and the residual graft material 30.1%.

**Figure 7:**
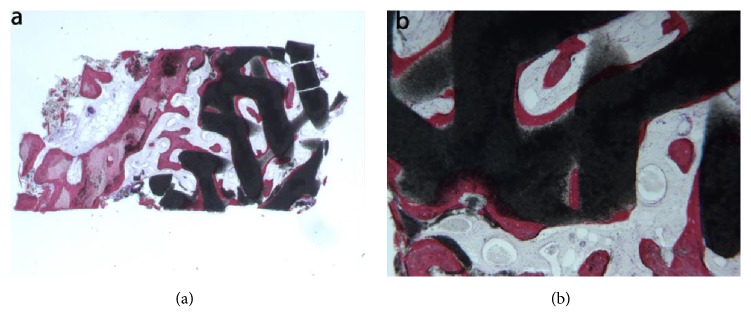
Synthetic micro-macroporous biphasic calcium-phosphate (HA-*β*-TCP 30/70) (toluidine blue and acid fuchsin): (a) trabecular bone with marrow spaces and residual biomaterial, located in the apical portion of the sample, could be observed (original magnification 12X); (b) the residual biomaterial was surrounded by newly formed bone and no gaps were present at the bone biomaterial interface. In some fields, the graft seemed to undergo resorption. Many large blood vessels could be seen (original magnification 40X).

Synthetic micro-macroporous biphasic calcium-phosphate (HA-*β*-TCP 30/70). In the examined sample, newly formed trabecular bone and preexisting bone with marrow spaces and residual biomaterial could be observed (Figure 7(a)). At low power magnification, the residual biomaterial was surrounded by newly formed bone and no gaps were present at the bone biomaterial interface. In some portions of the specimen the graft seemed to undergo resorption (Figure 7(b)). No inflammatory cells or multinucleated giant cells were present around the biomaterial or at the interface with bone. Many small and large sized vessels could be observed. Histomorphometry showed that newly formed bone represented 20.3%, marrow spaces 41.8%, and the residual graft material 37.9%.

**Figure 8:**
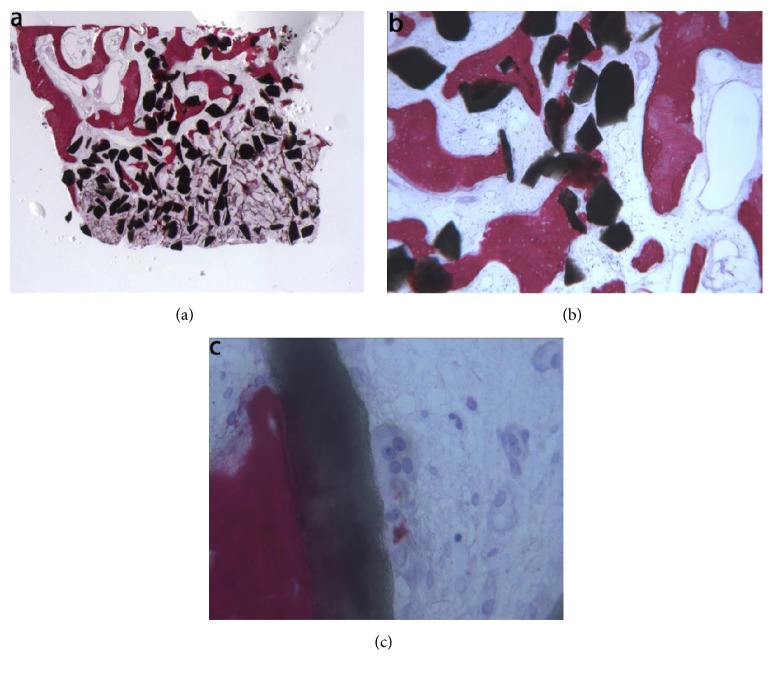
Bioapatite-collagen (toluidine blue and acid fuchsin): (a) trabecular bone with marrow spaces and residual biomaterial particles was observed (original magnification 12X); (b) osteoblasts were observed in the process of apposing bone directly on the particle surface. Marrow spaces were colonized by small and large blood vessels in close proximity to the new bone and to the particles (original magnification 40X); (c) moderate inflammatory infiltrate and multinucleated giant cells, probably osteoclasts, were observed directly on the biomaterial particles surface (original magnification 400X).

Bioapatite-collagen (BC). In the examined sample, trabecular bone with marrow spaces and residual biomaterial was observed (Figure 8(a)). Specifically, half of the sample was formed by residual biomaterial surrounded by newly formed bone, while in an apical portion of the sample many particles were partially covered by connective tissue. The new bone produced a network, ‘‘bridging” between the particles (Figure 8(b)). In a few fields, osteoblasts were observed in the process of apposing bone directly on the particle surface. Marrow spaces were colonized by small and large blood vessels in close proximity to the new bone and the biomaterial particles. Moderate inflammatory infiltrate and multinucleated giant cells, probably osteoclasts, were observed directly on the biomaterial particles surface (Figure 8(c)). Histomorphometry showed that newly formed bone represented 21.4 %, marrow spaces 53.3%, and the residual graft material 25.3%.

The histomorphometric results of bone biopsies are summarized in Table 2.

## 4. Discussion

Sinus augmentation is a well-documented technique for creating adequate bone volume to successfully place dental implants in resorbed maxillary posterior regions [36]. However clinical and histological outcomes regarding bone substitute materials still remain open areas of investigation because an ideal grafting material should provide biologic stability, ensure volume maintenance, and induce a high rate of formation of vital bone and bone remodeling [19]. Although numerous studies have compared grafting materials after sinus augmentation [7, 9, 16, 19, 27, 37–39], no one has compared histological and histomorphometric results of MCBA, FDBA, ABB, EB, HA-TCP (30/70), BC, using a standardized two-stage sinus augmentation model.

Within the limits of the present investigation, whose results referred to a limited number of patients, the histological and histomorphometric analysis of the regenerated tissues might provide useful information regarding the nature and amount of newly formed bone of the six tested biomaterials.

At histologic examination all biomaterials were in close contact with newly formed bone and showed the same pattern of bone formation surrounding the graft granules and producing a bridge-like network between the grafted particles.

From the present histologic investigation, the MCBA sample showed the highest biocompatibility, because no signs of acute inflammation were present, which was furthermore affirmed by the ability to form and maintain new bone bridging the greatest part of the biomaterial particles, as reported by other previous studies [9, 22, 38]. The use of MCBA tends to result in a slightly lower level of new bone formation compared to autologous bone, even if this tendency was not significant in a meta-analysis [3]. At the histomorphometric examination the percentage of new bone (20.1%) was lower than the percentage found by Schmitt et al. (35.41%) [8], while the residual biomaterial (22.4%) was comparable to the value reported for other graft materials [40]. Moreover, our results agreed with histologic examination of NOUMBISSI et al. [41], in which the graft turnover (resorption and replacement by new bone) occurred more rapidly in MCBA.

Histomorphometry of FDBA sample showed that newly formed bone represented 32.1%, the highest value among the compared biomaterials. This data was similar to results of Kolerman et al. [42], who reported 27.5%, but lower than 41.1% described by Cammack et al. [43] and confirmed the capability to form a larger volume of bone in shorter times during clinical trials, as reported by other investigations [10, 44]. Sbordone et al. found that FDBA had similar outcomes compared to autogenous bone in sinus grafting procedure even when the residual floor thickness was less than 3 mm [45]. Furthermore, the evidence of resorption phenomena in our histology confirms that this material could influence long-term results in regenerated sites [23].

ABB was used as grafting material in a great number of studies, taking advantage of its well- known osteoconductive properties [44]. Some authors showed that microvascular density at 6 months in sinus augmented with ABB was not significantly different from microvascular density in sites augmented with autogenous bone [46]. In the present investigation ABB sample showed areas of new bone formation in close contact with the biomaterial surface and no signs of inflammation, suggesting a neutral interaction of the grafted particles with the new bone tissue. Moreover, compared to the other biomaterials at the histomorphometric examination, ABB showed the lowest percentage of newly formed bone (16.1%) and a higher amount of remaining biomaterial (37.2%). This no homogenous bone structure could, avoiding bone resorption, guarantee long-term stability of the augmented maxillary sinus [8]. Indeed, Mordenfeld et al. [15] showed long-term maintenance of these results after 9 years and Traini et al. [47] after 11 years.

Compared to ABB, EB showed a greater amount of newly formed bone (22.8%) and lower residual graft material (30.1%), in accordance with the results of a randomized clinical trial, which evaluated samples harvested 6 months after sinus augmentation with both of these materials [29]. This higher resorption could be influenced by a deantigenation process to which EB is subjected.

The presence, observed in our sample, of new bone surrounding the biomaterial particles, of many large vessels and in some fields of osteoblasts apposing bone directly on the particle surface is comparable to other studies [27, 47]. The ability of EB to achieve a more rapid and intense vascularization could promote long-term implant osseointegration and predictability of rehabilitation in regenerated sites.

The HA-*β*-TCP 30/70 blocks used in the present investigation had a reticular structure, manufactured by a rapid prototyping (RP) technique offering a precise control of the porosity and external shape of a Ha-TCP ratio ceramic bone substitute [16] so as to influence bone formation.

Indeed, the use of HA-*β*-TCP 30/70, whose degradation can be tailored by varying its chemical composition together with the incorporation of pores, seems to be a good strategy to overcome the low degradation rate of CaP ceramics, which represents a limitation of these materials [16].

On 3D reconstruction and quantitative analysis, the HA/TCP scaffolds exhibited good performances in terms of both bone regeneration and vascularization, independently of the specific scaffold morphology (i.e., granules or blocks) [47]. However, Giuliani et al. reported that the scaffold morphology could influence the long-term kinetics of bone regeneration by showing that block-based specimens presented better results than granule-based samples [48]. The data of the present study is in agreement with other investigations concerning bone formation in maxillary sinus augmentation with HA-beta-TCP (30/70), after a healing period of 6 months [34, 37].

The histological and histomorphometric aspects of BC sample were similar to those of the other materials tested and confirm the osteoconductive property of this biomaterial as shown in previous studies [34, 35]. Moreover, the plastic and spongy consistency of this biomaterial renders it very easy to handle and to shape with scissors, allowing it to be used in sinus augmentation procedures without any membrane, in contrast to granular grafts, as suggested by some authors [49, 50]. Lastly the presence of collagen and glucosamine improves, respectively, fibrin formation and bone mineralization process as confirmed by Maiorana et al. [51, 52].

## 5. Conclusion

Within the limitations of the present investigation, all the six biomaterials tested in two-stage maxillary sinus augmentation model showed good biocompatibility and osteoconductive properties and could be used successfully in sinus augmentation procedure. Although, the FDBA seemed to have the best histomorphometric result in terms of newly formed bone and residual graft material. Nevertheless, longer term histological studies will be needed to understand better resorption times and modalities [53, 54].

## Figures and Tables

**Table 1:** Patient characteristics at study baseline.

**N**	**Sex**	**Age**	**Implant location**	**Type of implant**	
				**Length**	**Diameter**
**1**	M	72	1.4	10	3.75
			1.6	11.5	5

**2**	F	62	2.6	10	5
			2.7	10	5

**3**	F	54	1.4	13	5.5
			1.6	13	5.5

**4**	F	50	2.4	10	4.3
			2.6	13	5.5

**5**	M	57	1.3	13	4
			1.5	10	4
			1.7	11.5	5

**6**	M	63	2.4	13	4
			2.6	10	5

**Table 2:** Histomorphometric results of bone biopsies retrieved from sinuses augmented.

	MCBA (%)	FDBA (%)	ABB (%)	EB (%)	HA-*β*-TCP 30/70 (%)	BC (%)
Newly formed bone	20.1	32.1	16.1	22.8	20.3	21.4
Marrow spaces	57.5	47.8	46.7	47.1	41.8	53.3
Residual graft material	22.4	20.1	37.2	30.1	37.9	25.3

MCBA: mineralized solvent-dehydrated bone

FDBA: freeze-dried mineralized bone allograft

ABB: anorganic bovine bone

EB: equine-derived bone

HA-*β*-TCP 30/70: synthetic micro-macroporous biphasic calcium-phosphate

BC: Bioapatite-collagen.

## Data Availability

The histological data used to support the findings of this study are included within the article.
